# Nanomechanical and Optical Properties of Anti-Counterfeiting Nanostructures Obtained by Hydrogel Photoresist in Laser Processing

**DOI:** 10.3390/biomimetics10120829

**Published:** 2025-12-11

**Authors:** Wei Wu, Qingxue Deng, Yuhang Shi, Jiyu Sun

**Affiliations:** 1 School of Mechanical Engineering, University of Shanghai for Science and Technology, Shanghai 200093, China; 254171399@st.usst.edu.cn (Q.D.); 2235050420@st.usst.edu.cn (Y.S.); 2 Key Laboratory of Bionic Engineering (Ministry of Education, China), Jilin University, Changchun 130022, China; sjy@jlu.edu.cn

**Keywords:** hydrogel photoresist, angle dependent color changing, anti-counterfeiting nanostructures

## Abstract

The microstructures of living creatures are widely used in bionics, and some can generate structural colors on biological surfaces and enable the process of dynamic camouflage. This study presents the hydrogel photoresist synthesized by polymerizing HEMA and MMA in THF solvent with initiator AIBN. Then, nanostructured gratings were fabricated on the hydrogel photoresists via double-beam interference lithography, and were characterized by scanning electron microscopy, angle-resolved spectroscopy system, and nanoindentation for pattern characterization, and nanomechanical and optical performance, respectively. Under multi-angle incident light, the optical computation of gratings with different depths indicates that a shallow implicit grating does not affect its dynamic color-changing performance. It is established that the laser power of 500 mW, a first exposure time of 5 s, and a second exposure time of 3 s are feasible for achieving efficient anti-counterfeiting nanostructures. The L_500-5-3_ has greater *Er* and *H* than that of L_500-5_ with the second processing, but smaller than ineffective patterns. And the depth of anti-counterfeiting gratings that is less than 0.8 μm is conducive to obtaining anti-counterfeiting gratings with different size parameters. The acquired anti-counterfeiting nanostructures exhibit excellent stability, reliability, and angle-dependent color changes under room light, which provides promising applications for security materials in daily life, sensors, optics, and electronics.

## 1. Introduction

Living creatures, such as chameleons, beetles, butterflies, plants, and birds, have evolved and produced various nanostructures, generating a variety of colors [1]. The surface structural color of their skin can present a variation process of dynamic camouflage by changing the lattice constant of microscopically ordered structures in vivo, which provides a promising way for information encryption and anti-counterfeiting information [2]. Squid and cuttlefish are renowned for rapid adaptive coloration that is used for a wide range of communication and camouflage through structural coloration, which plays a key role in augmenting their skin patterning [3]. The superficial thick layer of dermal iridophores of *Furcifer pardalis* is superimposed with a layer of guanine crystals and iridophore crystals, which enables this organism to camouflage and display brilliant colors [4]. Most biological materials are complex composites whose optical and mechanical properties are often outstanding, and the principal organic building blocks in living organisms are proteins, which are the main components for soft tissues [5]. Biological materials are difficult to imitate and are produced through artificial processing. Polymeric hydrogel has smart networks and exhibits some physical properties that are similar to biological material, which can change molecular interactions at critical onset points and have the ability to swell and shrink when physical stimuli change, including light, pressure, temperature, electric fields, magnetic fields, mechanical stress, and the intensity of various energy sources [6]. And some hydrogels can exhibit color changes when exposed to external stimuli, which has been used for information encryption/decryption with applications of monochrome, polychrome, white-light-emitting anti-counterfeiting, and other multi-dimensional anti-counterfeiting in military, pharmaceutical, biosensor, agriculture, and cosmetics fields [7,8].

Nowadays, anti-counterfeiting technology has been widely used in security labels for verifying the genuineness of products, such as counterfeit products (forged banknotes, artworks, brands, and individual documents), financial transactions, or military products [9,10,11]. And various anti-counterfeiting strategies have been explored, including watermarks, holograms, luminescence, barcodes, and two-dimensional codes [12,13,14,15]. However, traditional anti-counterfeiting strategies like watermarks, barcodes, and two-dimensional codes are easily copied and do not effectively prevent counterfeiting [12]. Based on the structural color of nanostructures, anti-counterfeiting tags/labels have significantly increased and have long-term commercial value due to their high stability, the difficulty to imitate and copy with the unique angle-dependent colors, their inexpensive production, and their exceptional ease of perception [16,17]. Many methods have been used for manufacturing micro–nano structures for structural color patterns, such as inkjet printing [18], angle-independent photonic crystal [19], self-assembled photonic crystals by magnetic field force [20], gravity [21], electric field [22], inverse opal photonic crystal [23], electron beam etching [24], ion beam etching [25], laser lithography [26], imprinting technology [27], surface wrinkle technology [28], etc. [29]. Laser lithography is a patterning method that is a simple and quick process over a large area without using a mask, and it has been applied in directed nano-photonics and surface plasmon resonance or large-area membrane reflectors and anti-reflectors [30]. And laser techniques consist of laser holographic imaging, encrypted laser holographic imaging, and laser lithography [31]. In order to imitate and obtain the micro–nano optical structure with camouflage characteristics of biological materials, the hydrogel material has been processed by laser technology to obtain bionic anti-counterfeiting materials [16,32]. A facile laser-induced dual polymerization method has been developed to fabricate a reversible photonic hydrogel for the continuous and reversible colorimetric determination of pH variations [33]. Slanted Bragg stack structures of hydrogel films were obtained by a nanofabrication method of laser interference lithography and silver halide chemistry in a cost-effective and rapid process [34]. The inkjet-printed multiwavelength laser encoding and anti-counterfeiting were achieved under a biologically programmable laser, in which the lasing can be manipulated by biomolecular activities at the nanoscale [35]. The orientation of advanced anti-counterfeiting developments is to closely track the latest achievements of chemical synthetic materials, integrate the technical characteristics of other disciplines, develop encryption compounds with special functions, and develop in the direction of improving their intersection and synthesis [8]. The development of multicolor polymeric hydrogels provides a greater capacity and higher security for stored information, thus making these a better choice for anti-counterfeiting, while more novel technologies that are of low cost and high efficiency still need to be explored [7,8,36].

The mechanical properties of materials are crucial indicators that provide a reference and basis for predicting service life, evaluating quality and reliability, optimizing processing, and forming processes in the design and selection of engineering applications. Moreover, when the size of structures approaches nanoscale, the physical properties of micro–nanomaterials will be quite different due to the volume, surface, and quantum effects of nanoparticles [37,38]. A polydimethylsiloxane/photonic crystal/polydimethylsiloxane sandwich structure was designed as a free-standing structural colored film, which can also be easily patterned by spraying methods and through embedding as a trademark on clothes with good durability and brilliant color [39]. The birefringent cellulosic film for eco-friendly anti-counterfeiting owns a complete shape and exhibits no obvious damage, even after being treated with ultrasonics to simulate the destruction of external forces [40]. For anti-counterfeiting materials, it is mainly focused on fabricating micro–nano structures, which have become one of the most competitive and promising technologies. However, there are few studies on the mechanical properties of anti-counterfeiting nanomaterials.

In this study, optical nanostructures were fabricated with a hydrogel of poly(HEMA-co-MMA), which serves as the raw material for preparing photoresist films for laser processing. And nanomechanical properties of anti-counterfeiting nanostructures were investigated to reveal their stability and reliability. Patterns appear as different structural colors at different observational angles under lighting, which makes them potentially useful as security materials in sensors, optics, electronics, and so on.

## 2. Materials and Methods

### 2.1. Materials

General hydrogel materials have the problem of being difficult to process by laser. The heat generated by the laser processing of hydrogels makes effective manufacturing more difficult. The thermal effect may cause unnecessary deformation of the hydrogel by triggering its thermal response characteristics or generating bubbles [41]. The hydrogel in this study was polymerized by HEMA and MMA (Chemically pure, Aladdin Biochemical Technology Co., Ltd., Shanghai, China), which can better resist the damage caused by the high heat of a laser. Poly(2-hydroxyethyl methacrylate) [P(HEMA)] is one of the most extensively studied hydrogels in biomedical applications, with a thermoset that is not enzymatically degraded or hydrolyzed by acidic or alkaline solutions [42,43]. Methyl methacrylate (MMA) is a monomer of acrylic resin that can be widely used in a variety of medical, dental, and industrial applications, and has been used as exterior latex paint for surface coating, in which the monomer is used most widely [44]. The HEMA-co-MMA copolymer, composed of HEMA and MMA monomers, stands out for its promising attributes and is suitable for precision optical and microelectronic processes. The IC semiconductor single crystal polished silicon wafers used in processing are prime grade (Prime, Lijing Silicon Materials Co., Ltd., Quzhou, China). The materials are chemical pure and can be directly used as received to prepare photoresist films, including azobisisobutyronitrile(AIBN, Chemical pure, Aladdin Biochemical Technology Co., Ltd., Shanghai, China), tetrahydrofuran (THF, Chemical pure, Aladdin Biochemical Technology Co., Ltd., Shanghai, China), and triarylsulfonyl hexafluorophosphate (Photoinitiator 6992, Sigma-Aldrich, St. Louis, MO, USA).

**Figure 1:**
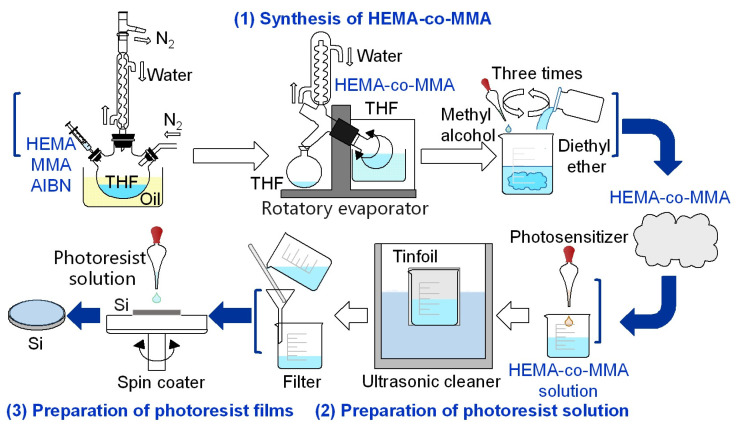
Scheme of photoresist films.

Synthesis of hydrogel: The 2-hydroxyethyl methacrylate (HEMA) -co-methyl methacrylate (MMA) was successfully obtained through chemical synthesis, providing the raw materials for spin-coating, and then for the final nanosecond laser with two-beam interference processing. The hydrogel is obtained from the experimental raw materials, which primarily consist of HEMA and MMA as the monomers, AIBN as the polymerization initiator, and THF as the solvent. Figure 1 illustrates the chemical reaction for the synthesis of a hydrogel material and preparation of photoresist films.

Preparation of substrate: Single-crystal, polished IC semiconductor silicon wafers were selected as the substrates. The thickness of one photoresist layer is several tens of nanometers, and the total thickness of the photoresist is a few microns, which was formed on the substrate through the spin-coating method. Since purchased silicon wafers may be contaminated with particles and dust that will lead to an uneven distribution of the photoresist during spin-coating and affect light absorption and reflection during laser processing, it is necessary to clean the wafers prior to use. Firstly, the silicon wafers were cut into approximately 1 cm × 1 cm specimens using a silicon knife, and then they were rinsed with deionized water to remove debris and extrinsic impurities from the surface. Subsequently, the silicon wafers were placed in anhydrous ethanol and cleaned in a numerical control ultrasonic cleaner machine (KQ-600DE, Kunshan Shumei Co., Ltd., Kunshan, China) for 5 min. This ultrasonic cleaning process was repeated twice. Finally, the wafers were taken out with a tweezer and placed in a vacuum oven for later use.

Preparation of photoresist solution: Fully dissolved HEMA-co-MMA solution was mixed with 15 μL of photosensitizer (triarylsulfonyl hexafluorophosphate) in the darkroom. The mixture was wrapped in tinfoil and then vibrated and stirred in a numerical control ultrasonic cleaner for approximately 15 min to ensure uniform dispersion of the photosensitizer within the HEMA-co-MMA solution. This step is essential to avoid the uneven distribution of the photoreceptor agents, which will affect the exposure effect. The stirred photoresist solution was then filtered in the darkroom to remove impurities during preparation. The manufacturing process of photoresist films is shown in Figure 1.

Preparation of HEMA-co-MMA precursor films: After adjusting the rotation speed of the spin coater (KW-4A benchtop spin coater, Beijing Tongde Co., Ltd., Beijing, China), clean silicon wafers were placed on the sample stage in the darkroom. The stage was then locked, and the vacuum was applied to secure the wafers. The wafers need to be centered on the sample stage, and the photoresist was carefully dropped onto the center of the wafer. Then, spin-coating was performed for 1 min. There will be residual solvent in the photoresist, which needs to be completely volatile after drying. And a drying oven (POLISH-20, Precision and technological manufacturing Co., Ltd., Shenzhen, China) was used here. The spin-coated samples were placed on a 50 °C dryer for 4 min and removed for laser processing.

### 2.2. Optical Simulation

The finite-difference time domain (FDTD) method was employed to simulate four distinct structural models under light incidence at various angles. These models include the following: (1) a grating model with both pitch and depth of 1 μm; (2) a grating model with a line pitch of 1 μm and depth of 0.8 μm; (3) a grating model with pitch of 1 μm and depth of 0.5 μm; and (4) a grid model with a side length of 1 μm and depth of 0.05 μm. Each model was simulated under incident light at angles of 0°, 10°, 20°, 30°, 40°, 41°, 42°, 43°, 44°, 45°, 46°, 47°, 48°, 49°, 50°, 60°, 70°, and 80°. The analysis range of the reflection data is set to 380–800 nm, a wavelength range that corresponds to visible light.

### 2.3. The Laser Direct Writing Process

**Figure 2:**
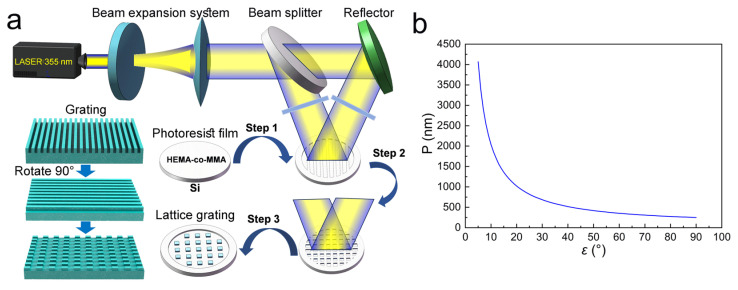
(**a**) Double-beam interference processing of a nanosecond laser; (**b**) the relationship between the grating period P and the incident angle *ε*.

The double-beam interference processing of a nanosecond laser is illustrated in Figure 2a. The beam emitted by the laser (Quanta-Ray Pro, Spectra-physics Newport, Newport Corporation Co., Ltd., Irvine, CA, USA) is first passed through the beam expansion system to optimize and adjust the spot size and quality, and then it is divided into two beams through the beam splitter. One beam is reflected by the semi-reflective lens, and the other beam is reflected by the reflector through the semi-reflective lens, and then the two beams meet with interference. The samples were placed at the position where the two beams intersect. The quality of the grating is improved by adjusting the size of the light intensity, the exposure time, and the energy. The exposure time is controlled by the closure of the valve, and the energy of the laser is adjusted by the handheld device. After the first exposure, the samples are rotated 90° for the second exposure to form a two-dimensional lattice structure. The wavelength of the laser is 355 nm. The maximum exposure power is 1 W, and the minimum exposure time is 1 s.

### 2.4. Measurement and Characterization

The samples were placed on stage using a scanning electron microscope (SEM, JEOL JSM-6700F, Thermo Fisher Scientific Co., Ltd., Waltham, MA, USA) to observe the architecture of the nanostructures. The optical characteristics of anti-counterfeiting materials were obtained via an angle-resolved spectroscopy system (ARM, IdeaOptics Instruments Co., Ltd., Shanghai, China) furnished with a halogen light source of 100 W. The incident light emitted on the samples is set to vary from 0° to 60° with an interval of 5°. The diameter of the light spot is about 50 μm. For nanomechanical testing, nano-indentations were accomplished at the surface of anti-counterfeiting nanostructures with a Berkovich tip. The parameters of the trapezoidal loading function were applied as follows: peak load = 500 μN, loading rate = 100 μN/s, and holding time = 5 s.

## 3. Results

### 3.1. Processing Design and Simulation

The light wavelength and emission angles of light beams are essential factors to influence the overlapping areas of the light waves in interference processing. Moreover, the interference intensity will be formed differently when the phase difference of the interfering waves is different in overlapping areas. Therefore, the parameters of light wavelength and emission angle can determine the kind of patterns and structures formed by interference. In this study, the light wavelength *λ* is 355 nm. The relationship between the incident angle ε and the grating period P in double-beam interference is as follows:

(1) $$P\;=\;\frac{\lambda }{2\sin\varepsilon }$$

The presentation of photolithography patterns is not only related to the optical system of laser processing, but it is also closely associated with the structural parameters of the processing [45]. The depth and shape of the grating directly influence the distribution efficiency of light energy across different levels, which is referred to as diffraction efficiency [46]. Sub-nanometer diffraction gratings (<1000 nm) are used for high-resolution spectroscopy, while medium step gratings are suitable for a wide spectral range with a 1000–10,000 nm micrometer. The size of grids larger than 1 micrometer can also interact with light to undergo diffraction, even exceeding the visible light band. However, the existence of the grid is visible to the naked eye, although the details of the structures cannot be seen. In order to obtain grating microstructures that are invisible to the naked eye, simulation analysis is conducted on grating models less than or equal to 1 μm to explore the relationship between the size of grids and optical performance. Thus, three depth grating models (1.0 μm, 0.8 μm, and 0.5 μm) and a grid model (0.5 μm) were developed for comparative analysis based on the manufacturing capacity of the laser processing equipment.

**Figure 3:**
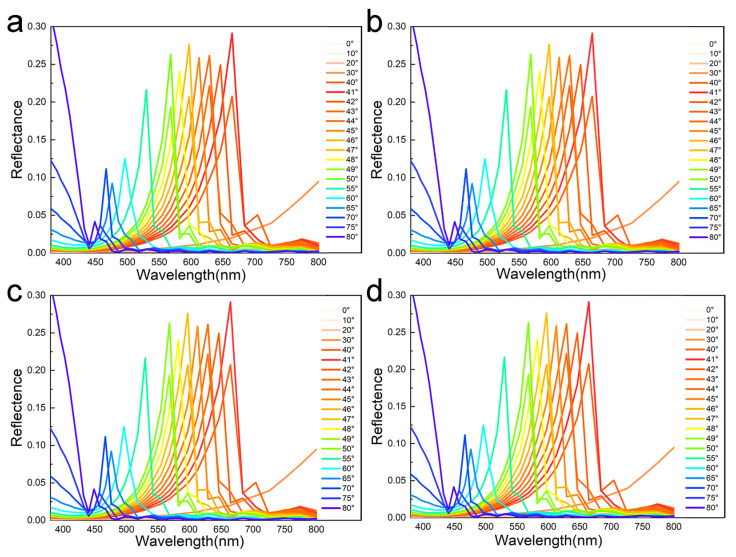
The reflectance of four grating models obtained by FDTD optical simulation: (**a**) the reflectance of the grid grating model with a depth of 1 μm; (**b**) the reflectance of the grid grating model with a depth of 0.8 μm; (**c**) the reflectance of the grid grating model with a depth of 0.5 μm; and (**d**) the reflectance of the lattice grating model with a depth of 0.5 μm.

To investigate the relationship between the optical performance of grid grating and lattice grating structures and their depth, optical simulation analysis was performed on four models using the finite-difference time domain (FDTD) method. The results of the simulations are presented in Figure 3. At incident angles ranging from 0° to 30°, no reflectance peaks are observed in the visible spectrum. The reflectance approaches 0 at the short wavelength (380 nm) and shows a monotonically increasing trend from 380 nm to 800 nm. As incident angles increase from 30° to 80°, all the reflectance curves exhibit a single distinct peak. The color of the model shifts from red under 40–41° to orange at 42–47°, yellow at 48°, green at 49–55°, blue at 60–65°, blue-purple at 65–75°, and purple at 80°.

### 3.2. Fabrication of Nanostructured Gratings

To ensure that the exposed grating pattern adheres perfectly to the substrate after development, it is necessary to prepare a photoresist film with a thickness of 1 μm. Then, the gratings will exhibit a period of approximately 1 μm after interference processing. Therefore, the required spin-coating speed needs to be explored to achieve the above photoresist thickness first. Figure 3 illustrates the relationship between the thickness of the photoresist and spin-coating speeds [47]. It is evident that as the rotation speed of the spin coater becomes faster, the photoresist film becomes thinner. When the spin coater operates at 1000 rpm, the thickness of the photoresist is 5.042 μm after drying, as shown in Figure 3a. It significantly exceeds the desired thickness for laser processing. Consequently, the speed of spin-coating should be increased. When the speed is adjusted to 3500 rpm, the photoresist thickness is reduced to 1.433 μm, as shown in Figure 3b, which approaches the desired thickness. With further fine-tuning, the photoresist thickness of 1.053 μm was obtained under the speed of 4000 rpm. The thickness of the photoresist films was prepared to achieve an appropriate grating period through laser interference processing. Finally, the spin-coating speed of 4000 rpm is selected for interference processing by a double-beam nanoscale laser.

When the laser processing is carried out, a chemical reaction will occur after absorbing a certain amount of light energy due to the addition of photosensitizers in the photoresist. And the degree of curing reaction differs as the light energy irradiated at different positions on the photoresist varies. This causes significant changes in the physical properties of the photoresist, such as solubility and affinity in the developer solution. The parts of the photoresist that can dissolve in the developer solution are removed; thus, the required pattern is obtained. Therefore, the photoresist can record the pattern of light intensity distribution that shines on its surface. Exposure dose is the most important parameter affecting photolithography. The integral expression is as follows:

(2) $$\;M\;=\;\int_{0}^{T}I_{1}\left(t\right)\mathrm{dt}\;$$

where *M* represents the exposure dose (mJ/cm^2^); *I*_1_ represents the intensity of exposure light (mW/cm^2^); and *t* is the exposure time (s).

The variables for controlling the laser energy output in this research are exposure power and exposure time. Therefore, Equation (2) is rewritten as the relationship between exposure dose, exposure power, time, and exposure area. The expression is as follows:

(3) $$M\;=\;\frac{U}{S_{1}}\;=\;\frac{W\;\times \;t}{S_{1}}$$

where *U* is the exposure energy (mJ); *S*_1_ represents the exposure area (cm^2^); and *W* represents the exposure power (mW).

Taking the exposure threshold dose of the commonly used SU-8 photoresist as a reference, the suitable exposure dose for the HEMA-co-MMA photoresist was investigated. According to previous studies, the exposure dose is set between 120 mJ/cm^2^ and 190 mJ/cm^2^ when the SU-8 photoresist is spin-coated with a thickness of 2 μm, and a smooth surface and uniform periodic patterns will be achieved by laser processing [48,49]. The maximum exposure power of the laser used in this study is 1 W, and the exposure area is constant at 1.6 cm^2^. Therefore, a higher exposure dose than the maximum exposure threshold value of 190 mJ/cm^2^ should be given to the HEMA-co-MMA photoresist.

**Figure 4:**
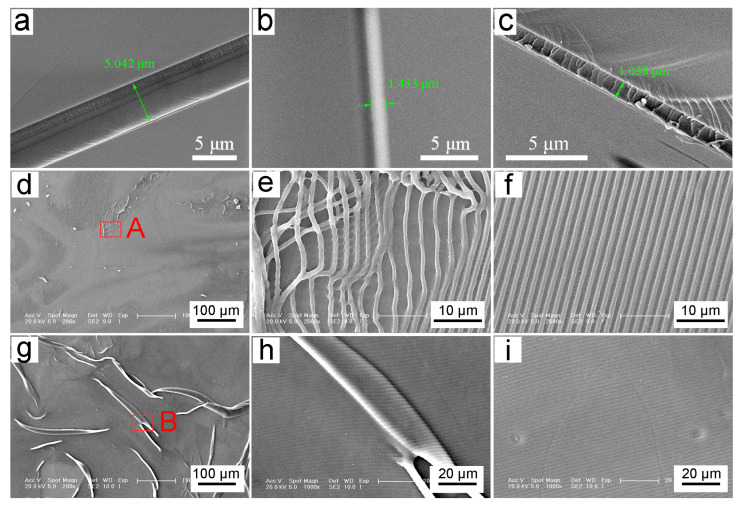
Thickness of photoresist at different spin-coating speeds: (**a**) 1000 rpm; (**b**) 3500 rpm; and (**c**) 4000 rpm. (**d**–**i**) Grating structures processed with different laser powers at the same exposure dose.

In order to obtain effective grating patterns, it is necessary to explore the exposure power and exposure time. Figure 4 demonstrates the results of different exposure powers on the exposure patterns of 1 μm-thick photoresist. Figure 4a–c show the design results when the exposure power is set to the maximum of 1 W and the exposure time is 1 s. According to Equation (3), the exposure dose on the HEMA-co-MMA photoresist is 625 mJ/cm^2^. The scanning electron microscopy (SEM) image of the exposed pattern at 200× magnification is presented in Figure 4a, revealing an uneven surface, and some areas are over-exposed. Area A exhibits distinct distortion, indicating overexposure. To better observe area A, it was amplified to 2500× magnification, as shown in Figure 4b. The exposed gratings detach from the silicon substrate and perform in a twisted state due to the extortionate laser power and high temperatures. Figure 4c gives the part of the grating structures with a smooth surface, exhibiting a grating period of 0.974 ± 0.036 μm, which consists of the theoretical grating period.

Figure 4d–f show the results obtained by an exposure power of 500 mW and an exposure time of 2 s. Figure 4d shows the exposed pattern at 200× magnification under SEM, revealing numerous folds in the exposed areas. For further observation, the fold area B was magnified to 1000×. It is found that the folded part has unbroken grating structures, and the structures are arranged neatly and evenly. Furthermore, the arrangement direction of the fold part is consistent with the surrounding pattern of the unfold part, as shown in Figure 4e. Figure 4f presents the pattern of the unfolded part with a grating period of 1.08 ± 0.04 μm, which is slightly larger than that of the theoretical period. However, there is no photoresist pattern remaining after exposure and development when the power is adjusted to 400 mW and the exposure time is increased to 2.5 s. This implies that both the lithography and non-lithography parts may be dissolved in the developing solution and then removed. The relationship between exposure power and pattern formation is further investigated. Then, the laser power was reduced to 300 mW and 200 mW while maintaining the same exposure dose of 625 mJ/cm^2^ to obtain patterned samples. However, there were no patterns, which suggests that laser power is a critical factor in achieving successful patterning. After repeated experiments with multiple parameter adjustments of lithography, it was found that the laser power of 500 mW is feasible for the uniform and smooth grating structures.

**Figure 5:**
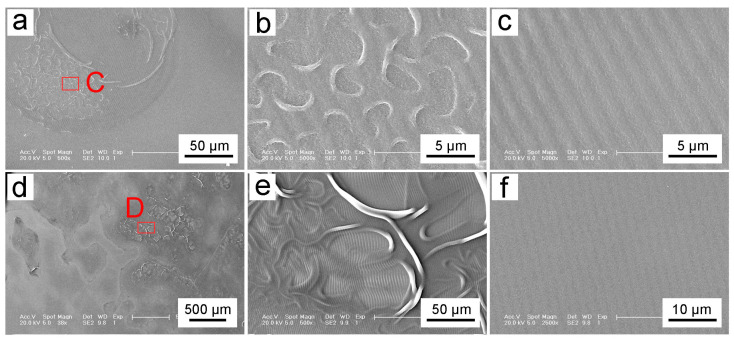
The grating structures processed under a laser power of 500 mW with different exposure times: (**a**–**c**) Damaged and fine grating structures under exposure time of 10 s; (**d**–**f**) Damaged and fine grating structures under exposure time of 7 s.

After a series of parameter studies under the same exposure dose with different exposure powers, the effect of different exposure times on the lithographic pattern at a laser power of 500 mW was further explored. Figure 5a–c present the photolithographic patterns resulting from an exposure time of 10 s. Figure 5a displays the pattern under SEM at 500× magnification. It reveals a part of the area with grating and non-grating structures. The grating structures are uniform and well-defined, but the rough areas have no grating structures. A further observation of rough area C was performed at 5000× magnification. And the photoresist in this area was exposed too much, which results in only breaks and shrinks, as shown in Figure 4b. Figure 4c shows the grating structures with a photolithographic pattern with a periodicity of 1.049 ± 0.100 μm. The depth of grating structures appears relatively shallow.

Figure 5d–f shows the photolithographic patterns obtained with an exposure time of 7 s. Figure 5d presents the pattern at 38× magnification. It is obvious that the pattern is not uniform, and the rough part is centrally distributed. The grating structures are mottled, which results from the strong laser power and the long exposure time. The grating structure was destroyed, but not completely destroyed, which leads to the poor partial uniformity of the grating structure. After magnifying the rough region D to 500×, it is observed that the formed grating structure has been partially burned. The burning process leads to deformed and folded areas where the grating structures still exist. Figure 5f shows the grating with a uniform structure period of 1.141 ± 0.099 μm. The depth of the uniform grating further decreases and is smaller than that of 10 s exposure.

**Figure 6:**
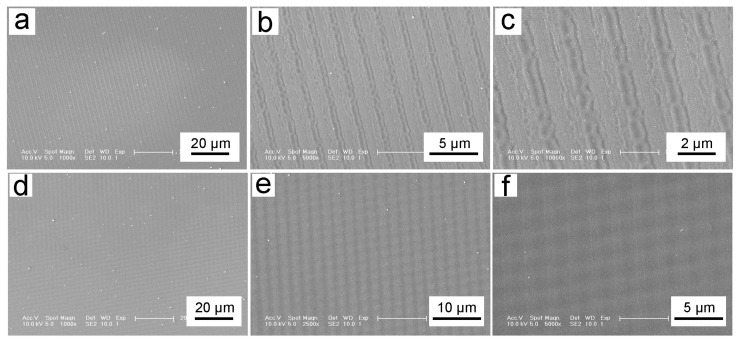
Anti-counterfeiting nanostructures: (**a**–**c**) Grating structures processed with a laser power of 500 mW and an exposure time of 5 s; (**d**–**f**) Two-dimensional square structure obtained by the secondary exposure time of 3 s.

With the above investigation, an exposure time of 5 s was applied, and the results of the photolithographic patterns are shown in Figure 6a–c. Figure 5a shows a uniform and ordered grating structure at 1000× magnification. In addition, there are no rough areas, as observed in Figure 6a,b. Therefore, the exposure time of 5 s is appropriate. Figure 6b and Figure 6c present the pattern structures under 5000× and 10,000× magnification, respectively. The depth of grating is shallower and performs a periodicity of 1.37 ± 0.05 μm. This may be due to the high laser power beam interference. The appropriate exposure time can help photoresist absorb light to quickly soften and cure, which causes the period of the grating structure to become larger. However, the grating structure obtained under this laser parameter performs at a high quality without the insufficient and rough parts of the deformed structure and fold. The samples obtained under this processing parameter not only have uniform and complete microstructures, but also have a smooth and flat surface when observed with the naked eye.

The above sample, processed with a laser power of 500 mW and an exposure time of 5 s, was rotated 90° for further exposure. Considering that 2 s exposure with 500 mW laser power will result in an uneven and wrinkled surface (as observed in Figure 6d), the exposure time of 3 s was chosen for the interference processing here. Figure 6d–f present the pattern at magnifications of 1000×, 2000×, and 10,000×, respectively. After the secondary exposure, the shape of the structure is square with the same size and well-arranged. And the length of the square is 1.55 ± 0.05 μm.

### 3.3. Nanomechanical and Optical Properties of Nanostructured Gratings

The properties were based on the observation results of the SEM images of the structural color-changing material samples processed by a nanosecond laser with dual-beam interference. Under the same exposure dose, when the laser processing power is 1 W and the exposure time is 1 s, the sample is named L_1000-1_. And the sample is named L_500-2_ when the laser processing power is 500 mW with an exposure time of 2 s. Other samples are named in the same way, such as L_500-10_, L_500-7_, and L_500-5_. When the L_500-5_ is subjected to secondary exposure for 3 s, the sample is named L_500-5-3_.

**Figure 7:**
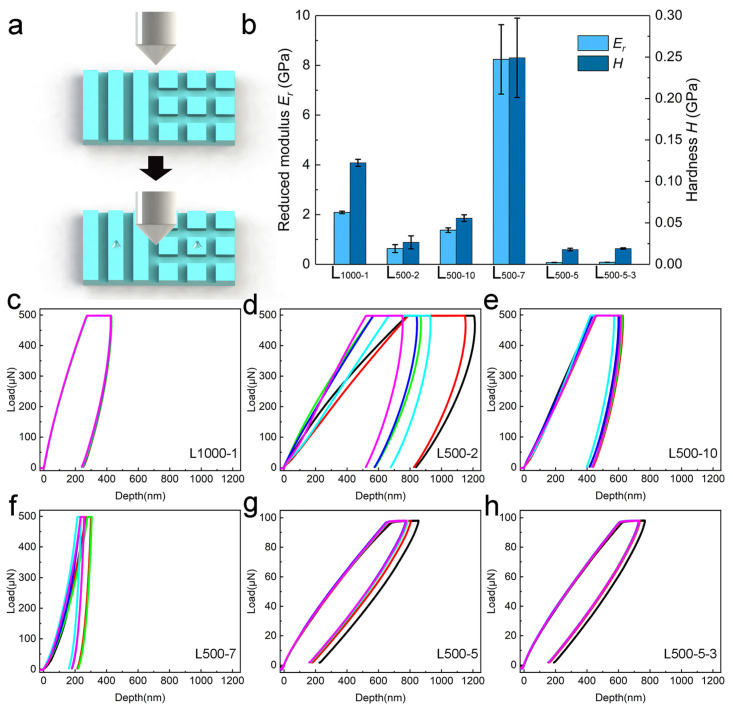
The nanomechanical properties of lithography specimens under different processing parameters: (**a**) A diagram illustrating the nanoindentation process and the indentation position; (**b**) The *Er* and *H* of six samples with different photolithographic patterns; (**c**–**h**) The displacement–-load curves for six samples.

Figure 7 shows the nanomechanical properties of the above samples. Experiments were conducted on the notches of the grating structures on the sample surfaces using a Berkovich tip, as shown in Figure 7a. Figure 7c–h show the displacement–load curves obtained from the experiments for each sample. The maximum load of 500 μN was applied to the overexposed samples, while the non-overexposed samples were tested under a lower load of 100 μN because of their shallower grating structures. And the displacement of the non-overexposed samples under the 100 μN was larger than that of the overexposed samples, which provides direct evidence of their differing mechanical properties. Additionally, the displacement–load curve for L_500-2_ exhibited a markedly higher degree of data dispersion. Through an analysis of the displacement–load curves for each sample, the *Er* and *H* of the biomimetic material are achieved by the Oliver–Pharr method [44], as shown in Figure 7b. The *Er* and *H* of the overexposed samples are larger than those of the non-overexposed samples. The *Er* and *H* of L_500-7_ are the largest, which are 8.241 ± 1.396 GPa and 0.249 ± 0.048 GPa, respectively, while the *Er* and *H* of L_500-5_ are the smallest, at 0.076 ± 0.004 GPa and 0.018 ± 0.002 GPa, respectively. The *Er* and *H* of L_500-5_ are 10.12% and 6.79% smaller than those of L_500-5-3_, respectively. For samples with poor microstructures, L_500-2_ and L_500-10_ perform with smaller *Er* and *H* than L_500-7_. And the *Er* and *H* of L_500-5-3_ are slightly higher than those of L_500-5_.

**Figure 8:**
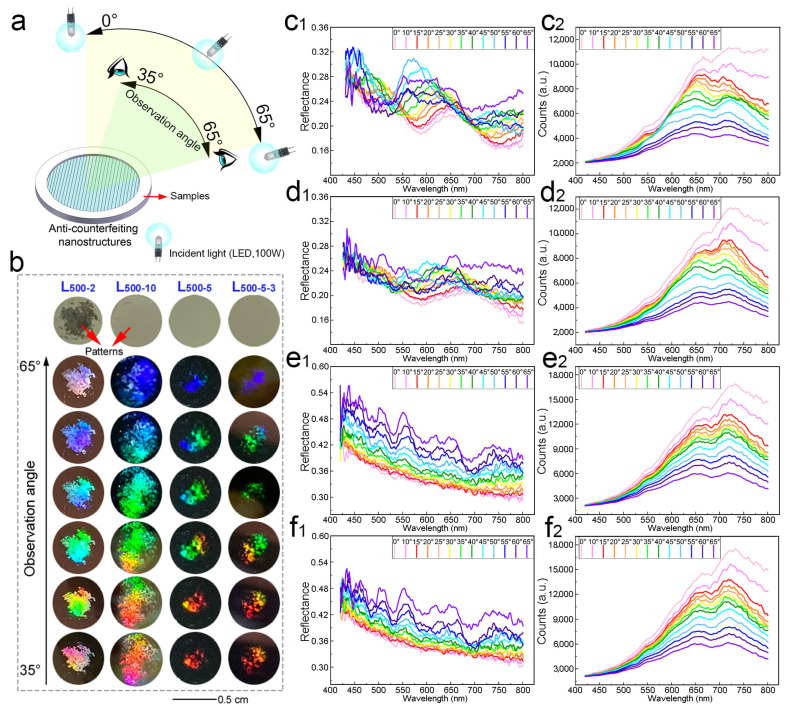
The optical properties of samples obtained by double-beam interference processing of nanosecond laser: (**a**) schematic diagram of optical testing under halogen light and observation under light-emitting diode; (**b**) angle-dependent color-changing of samples under light-emitting diode; (**c_1_**) the reflectance of L_500-2_; (**c_2_**) the relative light intensity of L_500-2_; (**d_1_**) the reflectance of L_500-10_; (**d_2_**) the relative light intensity of L_500-10_; (**e_1_**) the reflectance of L_500-5_; (**e_2_**) the relative light intensity of L500-5; (**f_1_**) the reflectance of L_500-5-3_; and (**f_2_**) the relative light intensity of L_500-5-3_.

With relatively regular surface microstructures and better color changing, the specimens of L_500-2_, L_500-10_, L_500-5_, and L_500-5-3_ were selected for comparison to the surface of unprocessed silicon wafers under megascopic observation, as shown in Figure 8b. The sample L_500-2_ (Figure 4d) was obtained by the same exposure dose as L_1000-1_, then the samples L_500-10_ and L_500-5_ were achieved with laser processing under 500 mW laser power for different exposure times (Figure 5a and Figure 6a), and finally, the sample L_500-5-3_ was processed by secondary exposure (Figure 6d). The silicon wafer surface of L_500-2_ has obvious photolithography patterns. The photolithography patterns left on the surface of the L_500-10_ silicon wafer are not obvious, but they are still visible to the naked eye. The silicon wafer surface of L_500-5_ has a grating structure, but it presents the same smooth state as the surface of unprocessed silicon wafers, and there are no visible photolithography patterns. After secondary exposure, the silicon wafer surface of the L_500-5-3_ sample changed from a grid structure to a lattice structure, but it remained smooth and had no visible photolithography patterns.

The above four photolithography samples were placed under LED spotlights (7070 series, Shanghai Yaming Lighting Co., Ltd., Shanghai, China), and multi-angle shooting was carried out with a camera (D7000, NIKON Co., Ltd., Tokyo, Japan), as shown in Figure 8a. All four photolithography samples could undergo multi-angle color changes across the entire wavelength range and could present multiple colors at the same angle (Figure 8b). When the observation angle increases from 35° to 65°, L_500-2_, L_500-10_, L_500-5_, and L_500-5-3_ change from red to orange, yellow, green, blue, indigo, and purple (Figure 8b). This is due to the different laser processing parameters, which result in different grating constants of the grating structure on the sample surface.

Figure 8c,d show the multi-angle optical test results of the L_500-2_ and L_500-10_. The reflectance value of L_500-2_ is relatively large in the 420–500 nm band. As the test angle increases from 0° to 60°, the peak position of its reflectance curve shifts from 665 nm to 552 nm, that is, from red to green. The color of the sample is the superposition of the color in the 552–665 nm band and the color in the 420–500 nm band. The sample shows multi-angle changes, and its color changes are purple, brown, and blue-green (Figure 8(c_1_)). As the test angle increases, the relative intensity of light reflection of the L_500-2_ sample decreases; that is, the number of photons received per second reduces (Figure 8(c_2_)). Therefore, as the observation angle increases, the light intensity received by the human eye will decrease. The reflectance curve of L_500-10_ is similar to that of L_500-2_. Its reflectance value is relatively large in the 420−500 nm band, with the peak located between 600 nm and 700 nm (Figure 8(d_1_)). As the test angle increases from 0° to 40°, the peak position of the L_500-10_ reflectance curve shifts from 654 nm to 601 nm. When the test angle is further increased, the peak position remains basically unchanged. The reflectance value increases from 24.7% to 26.5%, and the color of the sample shows multi-angle changes. The reflection intensity of light by the L_500-10_ also decreases as the test angle increases (Figure 8(d_2_)). Figure 8e,f show the multi-angle optical test results of L_500-5_ and L_500-5-3_. When the test angle is 0–30°, the reflectance curve of the L_500-5_ shows no obvious peaks, and the sample appears gray. As the test angle increases, the peaks become more and more distinct, and there are multiple peaks (Figure 8(e_1_)). The changing trend of the reflectance curve of L_500-5-3_ is similar to that of L_500-5_ (Figure 8(f_1_)). When the test angle is 0–30°, there are no obvious peaks. When the test angle increases from 35° to 60°, the peaks become more and more obvious, and there are multiple peaks (Figure 8(e_2_)).

## 4. Discussion

According to the results of optical simulation, when the incidence angle changed from 48° to 50°, the color changed in diversity, and the color changed every one degree. The angle-dependent color of models changes from red to green as the incident angle increases, which is exactly a result of the Bragg diffraction shift in periodic structures [50]. The value of the reflectance peak fluctuates between 30° and 50°, and it shows a decreasing trend while the incident angle is greater than 50°. When the incident angle is between 40° and 50°, the peak reflectance is in the interval of 20–30%, and then decreases to 5% and even smaller with a larger incident angle than 60°. The different depths and shapes of the models can perform almost the same optical properties for grating microstructures. Therefore, an implicit microstructure can be obtained by reducing the depth of the grid to achieve anti-counterfeiting functions and ensure optical performance of angle-dependent color-changing.

Consider the experimental results of nanoindentation, the marked data dispersion in the displacement–load curves for L_500-2_ indicates a rougher surface [51]. The laser parameters have an obvious impact on the mechanical properties of metals, photoresists, and polymers [52,53,54]. Processing power and exposure time will affect the cross-linking degree of photoresists during the photochemical reaction process, causing differences in their *Er* and *H* [55]. The *Er* and *H* of L_500-7_ are greater than those of L_500-2_ and L_500-10_. Therefore, it is not the case that the longer the exposure time, the greater the *Er* and *H* of the sample will be. The *Er* and *H* of L_1000-1_ are greater than those of L_500-2_. This implies that larger processing power can increase the *Er* and *H* of the samples under the same exposure energy of a laser. The *Er* and *H* of L_500-5_ are 10.12% and 6.79% smaller than those of L_500-5-3_, respectively. On the basis of the first exposure, a second exposure is carried out, and the photoresist is etched twice, resulting in higher *Er* and *H*. Compared with the influence of exposure time and processing power on the *Er* and *H* of the sample, secondary exposure can increase the *Er* and *H* of the sample, but the degree of its influence is relatively small. Appropriate processing power and exposure time will not cause significant changes in the *Er* and *H* of the sample, and the *Er* and *H* of the sample will be smaller than those of overexposed and underexposed samples. The nanomechanical properties of the prepared grating structures are close to those of the surface of beetle elytra [56]. The mechanical properties of the surface of the elytra can protect it well and resist damage from the external environment. The smaller *Er* and *H* of L_500-5-3_ and L_500-5_ indicate the ability to resist the destruction of microstructures with certain elasticity and hardness. The *Er* and *H* of L_500-5_ and L_500-5-3_ are lower than those of other samples, which indicates that these two samples are more prone to deformation when subjected to loads. However, lower *Er* and *H* indicate that the samples have better elasticity and can better dissipate the force exerted by external loads. And the structures of these two types of gratings are complete and smooth, indicating that they absorb and transfer energy more evenly during the laser processing stage. The high *Er* and *H* of other samples may be caused by structural defects after processing.

From the observation results on the sample surface, it can be known that when the laser processing power is 500 mW and the exposure time is 5 s, the “implicit” bionic structure of the color-changing material can be processed. This grid structure exists on the sample surface but is not visible to the naked eye. After a secondary exposure of 3 s, the grid structure transforms into a lattice structure and remains firm and “implicitly” present on the surface of the sample, which performs anti-counterfeiting features. According to the grating equation *h (sinα ± sinθ_m_) = mλ*, different grating constants can lead to different directions, in which the maximum values of light of different wavelengths appear after interference, and the colors presented in different directions are also different. At the same time, the diffraction of light by the grating can cause the sample to present multiple colors at a certain angle. Due to the fact that the areas of the photolithography patterns obtained from samples processed with different laser parameters vary after development, the area of the photolithography patterns obtained from overexposure is larger, while the grating structure of the anti-counterfeiting photolithography patterns obtained from non-overexposure is shallower. Therefore, the color development areas of the L_500-2_ and L_500-10_ samples are larger. The color development area of the L_500-5_ and L_500-5-3_ samples is relatively small.

The color changes obtained from the L_500-2_ and L_500-10_ tests are different from those displayed under LED lights. The light source used in the optical testing is a halogen light source (TH4-200, Olympus Co., Ltd., Tokyo, Japan). The differences in the brightness and penetration of the light source will affect the color development of the sample. Moreover, L_500-2_ and L_500-10_ are overexposed samples, with wrinkles and unevenness on their surfaces, which affects the test results. The reflectance curves obtained from thin film interference materials generally have multiple peaks. The reflectance curves of L_500-5_ and L_500-5-3_ are similar to them. Therefore, the depth of the grating and square array structures of the “implicit” bionic structure color-changing materials L_500-5_ and L_500-5-3_ processed by nanosecond laser dual-beam interference is shallow. It has a reflection and refraction effect similar to that of a single-layer film on light. When the test angle is 35–60°, both L_500-5_ and L_500-5-3_ simultaneously exhibit multiple colors. The larger the test angle, the more distinct the colors, which is basically consistent with the observed results. The relative intensity of light reflection of L_500-5_ and L_500-5-3_ decreases with the increase in the test angle (Figure 8e_2_,f_2_), which is the same as that of the overexposed samples. However, this does not prevent L_500-5_ and L_500-5-3_ from presenting colors. Meanwhile, the grating structure on their surfaces has a diffraction effect on light, which can form rainbow colors, making the reflectance curve have multiple peaks. And a depth of anti-counterfeiting gratings of less than 0.8 μm can be viable for obtaining anti-counterfeiting gratings with angle-dependent color-changing, such as in L_500-5_ and L_500-5-3_.

## 5. Conclusions

The research introduces an innovative approach to anti-counterfeiting nanostructures obtained by synthesizing hydrogel photoresist in laser processing. The relationship between the structural depth and optical performance of the grating structure is revealed through simulation, and it is further verified that an anti-counterfeiting grating structure with the same optical performance can be obtained by reducing the grating depth. By preparing appropriate photoresist films and calculating the energy of laser processing, the anti-counterfeiting nanostructures were obtained with the adjustment of processing parameters during the laser processing. The laser power of 500 mW and exposure time of 5 s are valid for achieving efficient nanostructures, even with the second exposure time of 3 s. The L_500-5-3_ and L_500-5_ present invisible existence under the naked eye, but show obvious angle-dependent color-changing similar to other inferior exposed samples. The L_500-5-3_ has greater *Er* and *H* than that of L_500-5_ with the second processing, and both *Er* and *H* of L_500-5-3_ and L_500-5_ are smaller than those of ineffective patterns. The obtained anti-counterfeiting nanostructures exhibit stability, reliability, and angle-dependent color changes under room light.

## Data Availability

The original contributions presented in the study are included in the article, and further inquiries can be directed to the corresponding author.
